# Autosomal dominant transmission of complicated hereditary spastic paraplegia due to a dominant negative mutation of *KIF1A*, SPG30 gene

**DOI:** 10.1038/s41598-017-12999-9

**Published:** 2017-10-02

**Authors:** Chong Kun Cheon, So-Hee Lim, Yoo-Mi Kim, Doyoun Kim, Na-Yoon Lee, Tae-Sung Yoon, Nam-Soon Kim, Eunjoon Kim, Jae-Ran Lee

**Affiliations:** 1 Department of Pediatrics, Pusan National University Children’s Hospital, Yangsan, Korea; 2 Research Institute for Convergence of Biomedical Science and Technology, Pusan National University Yangsan Hospital, Yangsan, Korea; 3 Rare Disease Research Center, Korea Research Institute of Bioscience and Biotechnology, Daejeon, Korea; 4 Center for Synaptic Brain Dysfunctions, Institute for Basic Science (IBS), Daejeon, Korea; 5 Department of Biological Sciences, Korea Advanced Institute of Science and Technology, Daejeon, Korea

## Abstract

KIF1A is a brain-specific anterograde motor protein that transports cargoes towards the plus-ends of microtubules. Many variants of the *KIF1A* gene have been associated with neurodegenerative diseases and developmental delay. Homozygous mutations of *KIF1A* have been identified in a recessive subtype of hereditary spastic paraplegia (HSP), SPG30. In addition, *KIF1A* mutations have been found in pure HSP with autosomal dominant inheritance. Here we report the first case of familial complicated HSP with a *KIF1A* mutation transmitted in autosomal dominant inheritance. A heterozygous p.T258M mutation in *KIF1A* was found in a Korean family through targeted exome sequencing. They displayed phenotypes of mild intellectual disability with language delay, epilepsy, optic nerve atrophy, thinning of corpus callosum, periventricular white matter lesion, and microcephaly. A structural modeling revealed that the p.T258M mutation disrupted the binding of KIF1A motor domain to microtubules and its movement along microtubules. Assays of peripheral accumulation and proximal distribution of KIF1A motor indicated that the KIF1A motor domain with p.T258M mutation has reduced motor activity and exerts a dominant negative effect on wild-type KIF1A. These results suggest that the p.T258M mutation suppresses KIF1A motor activity and induces complicated HSP accompanying intellectual disability transmitted in autosomal dominant inheritance.

## Introduction

KIF1A is a motor protein that is expressed exclusively in the brain and transports cargoes from the cell body to peripheral ends of neurites^1,2^. Its motor domain is located at the N-terminus of the KIF1A molecule and uses ATP as energy sources for its movement along microtubules^3,4^. KIF1A transports synaptic vesicle precursors as well as dense-core vesicles through the C-terminal pleckstrin homology (PH) domain that binds phosphoinositides with high affinity and specificity^5–8^. When cargo-containing lipid vesicles bind to the PH domain, the KIF1A motor becomes a dimer through coiled-coils interactions located in the immediate downstream regions of the motor domain and gets activated for movements along the microtubules. Although KIF1A has been thought as a monomeric motor unlike other kinesin families, lines of evidence suggest that KIF1A can be converted into functional dimer^9–12^. *KIF1A* null mice display severe motor and sensory disturbances, and die within a day or so after birth^5^. In addition, evidences from mice indicate that KIF1A regulates hippocampal synaptogenesis and learning enhancement^13^.

Various heterozygous de novo missense mutations on the KIF1A motor domain have been identified in patients who display intellectual disability along with atrophy and spastic paraplegia^14–17^. In addition, homozygous recessive mutations of *KIF1A* have been found in the hereditary sensory and autonomic neuropathy type 2 (HSANII) and in a recessive subtype of hereditary spastic paraplegia (HSP), SPG30^18–21^. More, recently, there have been two cases of autosomal dominant inheritance of *KIF1A* mutation; a de novo variant in the KIF1A motor domain (p.S69L) was transmitted dominantly from father to son with pure form of HSP, and the same heterozygous variant of *KIF1A* was identified in a family with a dominant segregation pattern of pure HSP^22,23^. Spastic paraplegia is characterized by progressive stiffness and contraction in the lower limbs. When additional symptoms or signs are present, the patients are diagnosed as ‘complicated’ rather than ‘pure’ spastic paraplegia. Complicated spastic paraplegia could be accompanied by systemic or neurologic abnormalities such as ataxia, seizures, intellectual disability, dementia, amyotrophy, extrapyramidal disturbance, or peripheral neuropathy^24,25^.

Here we report the first case of familial complicated HSP with a *KIF1A* mutation transmitted in autosomal dominant inheritance. This p.T258M mutation is located in the conserved sequences of the motor domain, and was identified from four patients in a family of HSP along with mild intellectual disability, language delay, optic nerve atrophy, thinning of corpus callosum, periventricular white matter lesion, microcephaly, and epilepsy. A structural modeling and assays using hippocampal neurons showed that this *KIF1A* mutation could bring about loss-of function of KIF1A motor and exert a dominant negative effect. It has been thought that the reduced reproductive fitness in intellectual disability could make an autosomal dominant inheritance uncommon^14^. Therefore, this novel heterozygous p.T258M mutation with autosomal dominant inheritance is meaningful in that it expands the phenotypic spectrum of *KIF1A* variants to include intellectual disability and spastic paraplegia.

## Results

### Clinical Characteristics of Family with heterozygous variant in *KIF1A*

**Figure 1:**
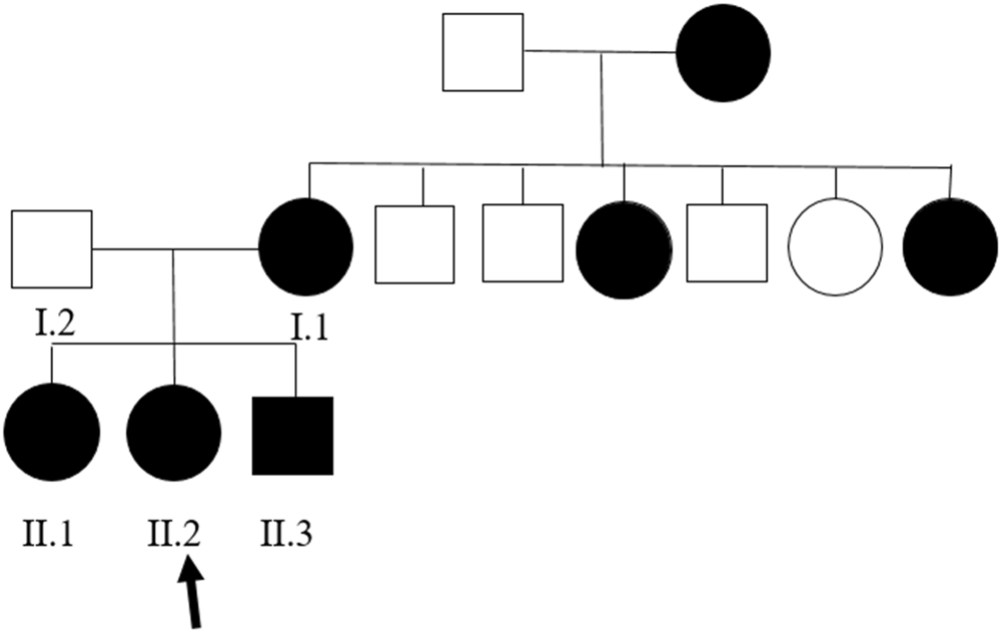
Familial segregation of the *KIF1A* variant. Pedigree structure of a family with c.773C > T (p.Thr258Met) variant in *KIF1A*. Black symbols represent patients with *KIF1A*. Arrow indicates proband. Only participants in the study for whom DNA is available for analysis are numbered.

**Figure 2:**
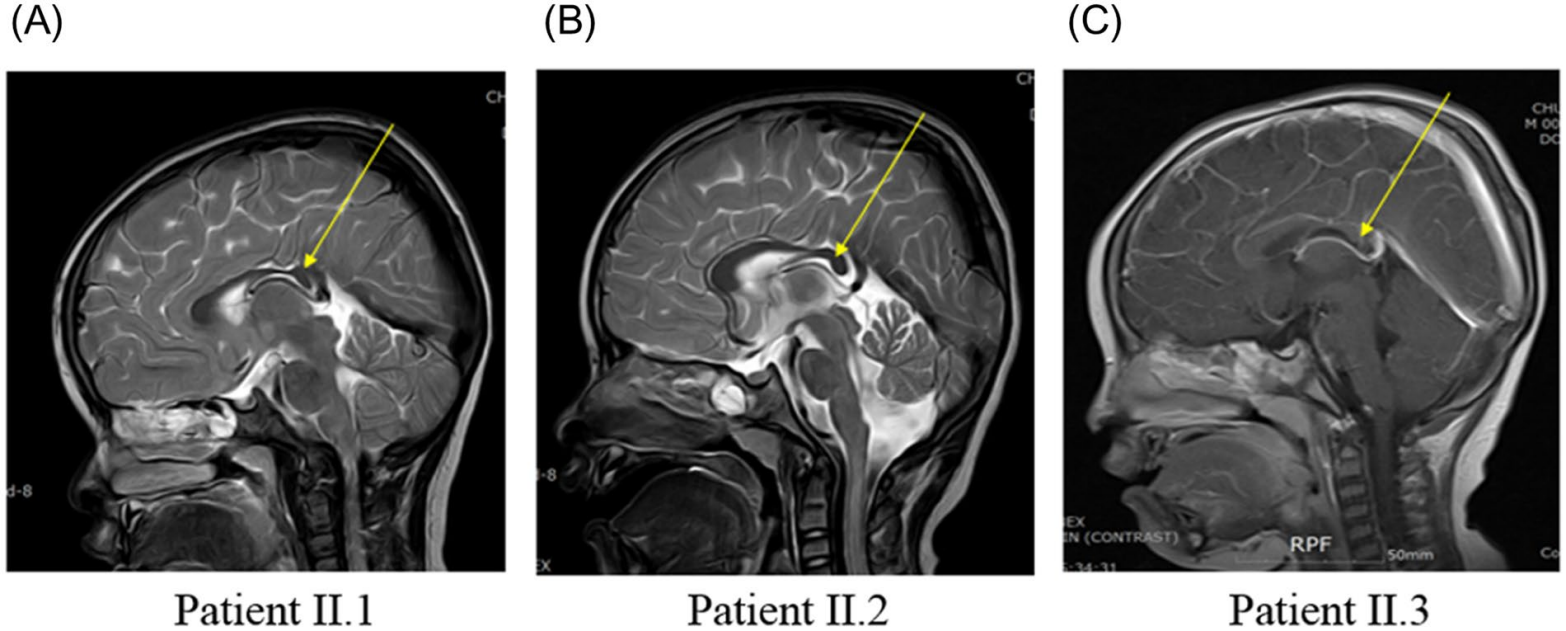
Axial T2-weighted magnetic resonance image of patients. (**A**, Patient II.1; **B**, Patient II.2; **C**, Patient II.3) MRI shows thinning of corpus callosum on axial T2- weighted image which is shown in yellow arrow.

**Figure 3:**
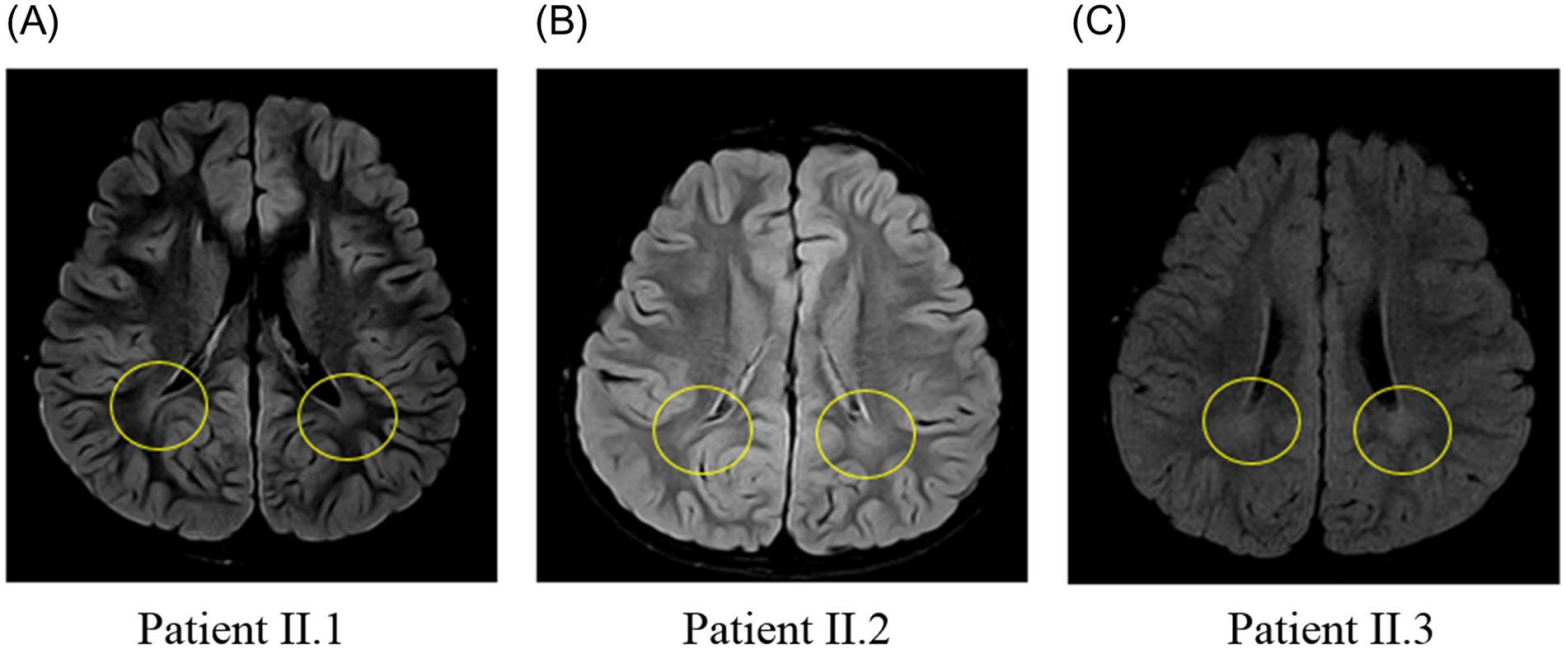
Fluid-attenuated inversion recovery (FLAIR) magnetic resonance image of patients. (**A**, Patient II.1; **B**, Patient II.2; **C**, Patient II.3) FLAIR imaging shows ill-defined lesion of periventricular white matter which is shown in yellow circle.

The pedigree for the family with *KIF1A* variant is shown in Fig. 1. All the enrolled family members were previously diagnosed with undiagnosed intellectual disability and spastic paraplegia. Clinical manifestations of patients with dominant *KIF1A* variant enrolled in this study were described in Table 1. Patient I.1, who was initially evaluated at the age of 35 years, is a woman from South Korea with mild cognitive impairment, optic nerve atrophy, and peripheral sensorimotor polyneuropathy affecting bilateral lower extremities. She underwent achilles tendon release operation due to spastic gait at age 17. On examination, she had both knee spasticity, Babinski reflex, and hyperreflexs on knees and ankles. She had increased tone and spasticity in the lower extremities with extensor plantar responses bilaterally. Family history is significant for her mother and two sisters with severe intellectual disability and spastic paraplegia. Patient II.1, who was evaluated at the age of 10 years, is a first daughter of patient I.1. She presented with mild cognitive impairment and spastic paraplegia. She was unable to roll over and had poor head control at the age of 6 months. She could walk alone at the age of 21 months. Her language development was delayed so that she only spoke a few words. She was then diagnosed with optic nerve hypoplasia at the age of 10 years. On examination, she had microcephaly (head circumference <3rd percentile) and her reflexes were hyperactive. Brain MRI revealed thinning of corpus callosum and ill-defined lesion of periventricular white matter (Figs 2 and 3). She showed thoracolumbar scoliosis. Patient II.2, who is a 7-year-old-girl with tip-toe walking, is a second daughter of patient I.1. She could walk first time when she was 18 months old. She was obese (body mass index 98.4th percentile). On examination, she has deformities on ankles, widening popliteal angle, and anterior pelvic tilting. She also showed moderate cognitive impairment and dysarthria. Reflexes were hyperactive and her strength was within normal limits for her age. Ophthalmologic evaluation showed pale optic disc. Brain MRIs showed thinning of corpus callosum and ill-defined lesion of periventricular white matter. Patient II.3, a 5-year-old-boy, had both equinus deformity and walked with spastic gait. He had both knees spasticity, Babinski reflex, and hyperreflexs on knees and ankles, and had great function with his hands. He developed partial seizure attack when he was 4 years old. Ophthalmologic examination showed pale optic disc. Brain MRIs showed thinning of corpus callosum and ill-defined lesion of periventricular white matter. He showed mild intellectual disability with speech delay.

**Table 1:** Clinical manifestations of patients with *KIF1A* variant enrolled in this study as well as a summary of clinical characteristics in the three previous reports of patients with de novo *KIF1A* mutations.

	Patient I.1	Patient II.1	Patient II.2	Patient II3	Nieh *et al*.^16^	Hotchkiss *et al*.^17^	Hotchkiss *et al*.^17^
*KIF1A* mutation	c.773 C > T (T258M)	c.773 C > T (T258M)	c.773 C > T (T258M)	c.773 C > T (T258M)	Various de novo missense mutations	c.595 G > A (G199R)	c.902 G > A (R307Q)
Gender	Female	Female	Female	Male	4 female, 2 male	Male	Male
Age (years)	35	10	7	5	1.5–16	6	14
Cognition & language	Mild ID with language delay	Mild ID with language delay	Moderate ID with language delay	Mild ID with language delay	Severe global developmental delay	Severe cognitive impairment with language delay	Severe cognitive impairment with language delay
Ophthalmologic involvement	Optic nerve atrophy	Optic nerve hypoplasia	Pale optic disks	Pale optic disks	3/6 Optic nerve atrophy 4/6 cortical visual impairment 1/6 abnormal eye movements, 1/6 cataracts	Nystagmus, pale optic disks	Pale optic disks
Brain MRI	nd	Thinning of corpus callosum	Thinning of corpus callosum	Thinning of corpus callosum	6/6 progressive cerebral and cerebellar atrophy	Thinning of corpus callosum, cerebellar Atrophy	Thinning of corpus callosum, cerebellar atrophy
Microcephaly	nd	Yes (<3rd percentile)	No (25th percentile)	No (10th percentile)	4/6 yes	No (25th percentile)	Yes (3rd percentile)
Contractures	Ankles(Achilles tendon release at age 17yrs), knees, elbow	Elbows, ankles, knees	Ankles, knees	Ankles	nd	Ankles(Achilles tendon release at age 6mo)	Elbows, ankles, knees
Spine	Scoliosis	Scoliosis	Normal	Normal	nd	Minor kyphosis	Scoliosis
NCV testing	Sensorimotor polyneuropathy in lower extremities	nd	nd	nd	nd	Axonal sensory-motor polyneuropathy	Distal motor neuropathy with absent sensory responses in upper and lower extremities
Epilepsy	no	no	no	yes	2/6 seizures 4/6 no seizures	No	yes

nd, not done.

### Genetic Study

**Figure 4:**
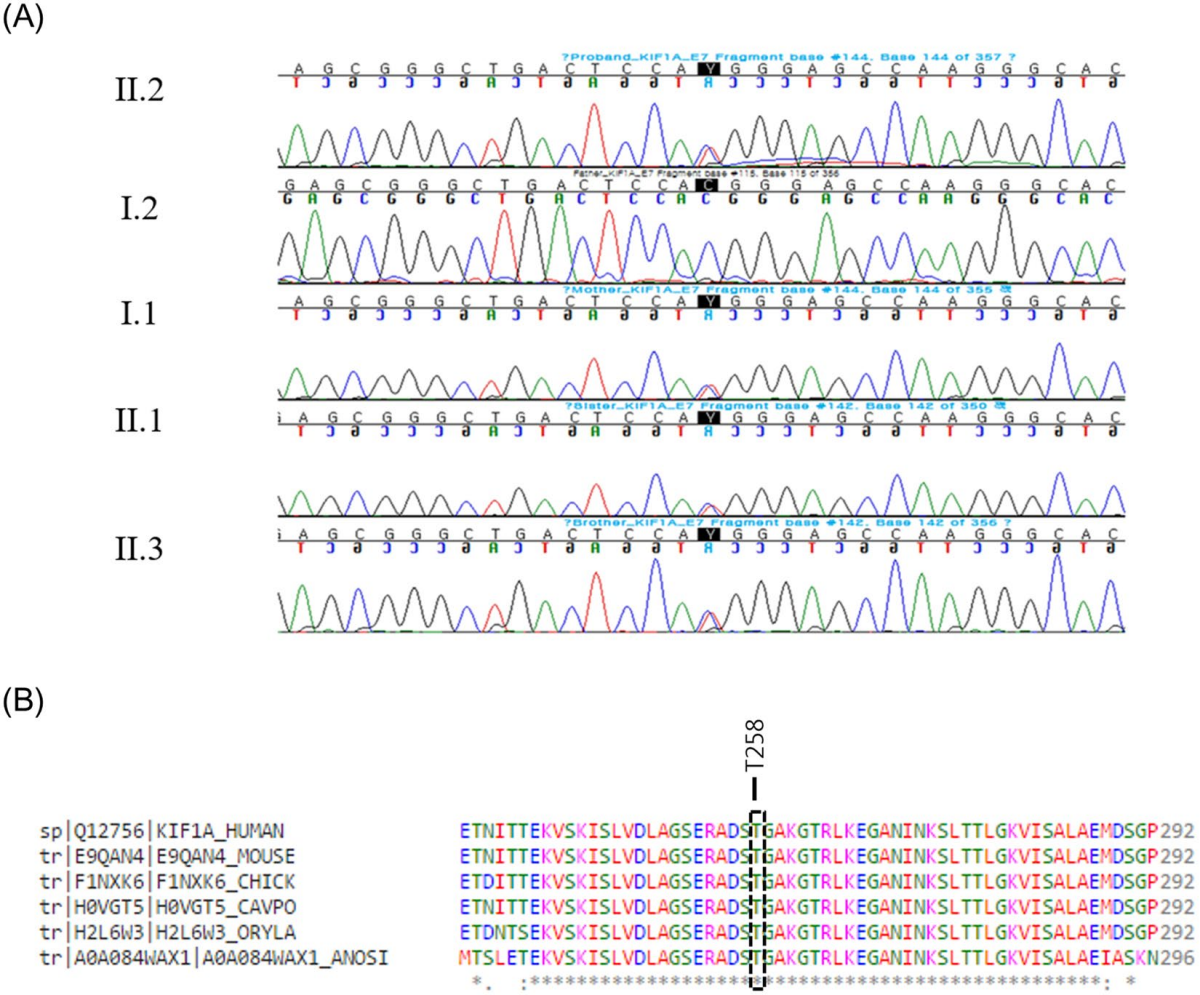
Pathogenic mutation identified in KIF1A. (**A**) Direct sequencing of the proband and her family shows a novel heterozygous mutation of *KIF1A* gene, c.773C > T (p.Thr258Met). (**B**) The p.T258 amino acid is completely conserved across available different species which is shown in black box. Multiple amino acid sequence alignment was generated using Cluster Omega (http://www.ebi.ac.uk/Tools/msa/clustalo) and includes human (Homo sapiens; Q12756; UniprotKB), mouse (Mus musculus; E9QAN4; UniprotKB), chicken (Gallus gallus; F1NXK6; UniprotKB), Guinea pig (Cavia porcellus; H0VGT5; UniprotKB), Japanese rice fish (Oryzias latipes; H2L6W3; UniprotKB), and Mosquito (Anopheles sinensis; A0A084WAX1; UniprotKB).

The study included 4 subjects from a non-consanguineous family presenting with cognitive impairment, spastic gait, and negative test result for the spastic paraplegia 4 (SPG4) gene. In the next step, targeted exome sequencing (TES) in the four patients was applied using the Nextseq. 500 sequencing platform and TruSight One Sequencing panel (Illumina, USA) to identify the genetic cause of undiagnosed intellectual disability and spastic paraplegia. Written consent was obtained from the family for the blood samples. TES results revealed heterozygous novel variant, c.773 C > T (p.Thr258Met), on *KIF1A* exon 7 which was confirmed by Sanger sequencing (Fig. 4A). This variant is predicted to be damaging according to *in silico* analysis (SIFT 0, PolyPhen2 0.983, MutationTaster 1). The variant was not detected in control population (1000 genomes, ESP 6500, and ExAC). The threonine at position 258 is a highly conserved amino acid residue among different species (Fig. 4B). Parental segregation was positive, indicating that the mutations occurred in a family.

### Structural impact of p.Thr258Met mutation on the motor domain of KIF1A

**Figure 5:**
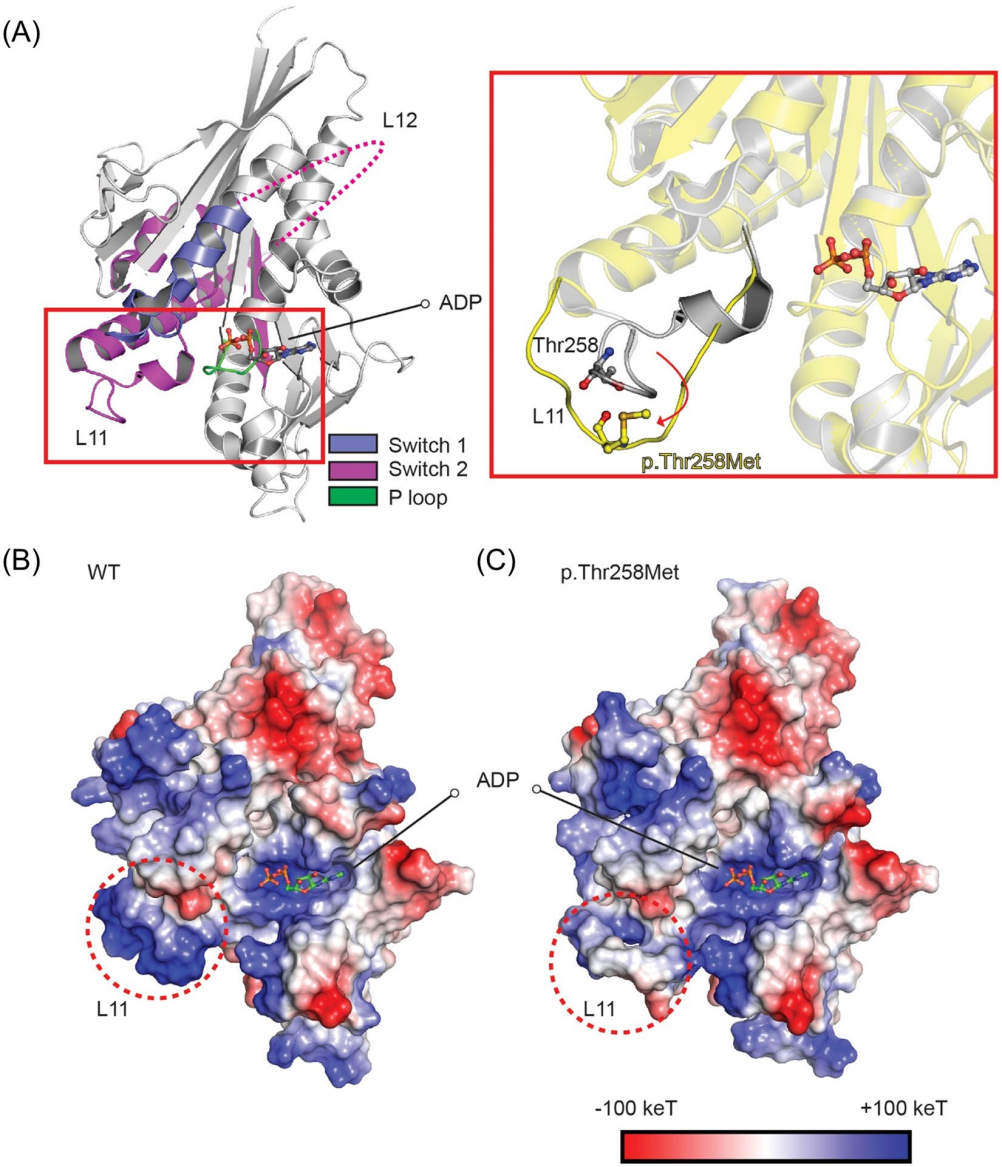
Structural modeling of the KIF1A motor domain with p.T258M mutation. (**A**) Modelled structure of the KIF1A motor domain mutant (p.Thr258Met). The crystal structure of the motor domain from WT KIF1A in complex with ADP [PDB ID: 1I5S] is depicted as a cartoon representation. The ATP binding loop (P-loop), switch-1, and switch-2 are shown in green, deep blue, and purple, respectively. L11 and L12 (unmodelled) in switch-2 are also indicated. The ADP molecule is shown by the ball-and-stick model. The red box indicates a close-up view of the boxed region. The structure of the modelled mutant (p.Thr258Met) motor domain of KIF1A is shown in yellow. The conformational change in L11 predicted to be induced by the p.Thr258Met mutation is indicated by a red arrow, together with the structure of WT L11 shown in grey. (**B,C**) Electrostatic charge distribution patterns in the WT (**B**) and modelled mutant p.Thr258Met (**C**) motor domains of KIF1A, as shown by the surface model. Negative and positive charges are shown in red and blue, respectively. The regions of charge changes (L11) are indicated by red dotted circles.

The crystal structure of the motor domain of KIF1A in complex with ADP and ATP analogues have been reported^26,27^. The results from these studies indicate that the binding and hydrolysis of ATP to the motor domain of KIF1A induce conformational changes in the motor domain that drive the movement of KIF1A along microtubules. The ATP-binding pocket in the KIF1A motor domain contains a P-loop (GX_4_GK[T/S]) and parts of the switch-1, and switch-2 regions (Fig. 5A). The binding and hydrolysis of ATP to the KIF1A motor domain lead to conformational changes in switch-1 and switch-2. These changes allow for the loop 11 (L11) and the loop 12 (L12) in switch-2 alternatively to bind to microtubules for KIF1A movement^28^. Therefore, L11 is important for ATP-dependent binding of KIF1A to microtubules for motor movement.

A structural modeling of the KIF1A motor domain containing the p.Thr258Met mutation showed that the point mutation leads to the disruption of the structure of L11 (Fig. 5A, red box). In addition structural alterations, the p.Thr258Met mutation caused changes in electrostatic charge distribution in L11, neutralizing the positive charges in L11, as shown by charge distribution patterns of wild-type (WT) and mutant motor domains of KIF1A (Fig. 5B,C). These results collectively suggest that the p.Thr258Met mutation disrupts both the conformation and electrostatic charge of L11 in the KIF1A motor domain, leading to the suppression of the binding of the mutant KIF1A protein to microtubules and hence causing limited motor movements along microtubules.

### Functional impact of p.Thr258Met mutation on KIF1A motor activity

When EGFP tagged motor domain of WT KIF1A (aa 1–365, KIF1A-MD-EGFP) was expressed in rat hippocampal neurons, it was dramatically accumulated in distal regions of the neurites in a large proportion of the transfected cells^10^. However, induction of a point mutation (p.T312M) that impairs the motor activity resulted a greatly reduced peripheral accumulation of KIF1A motor domain proteins^2,10^. Previously we used this functional assay to test the impact of de novo missense mutations of *KIF1A* identified in patients with cognitive impairment^15^.

**Figure 6:**
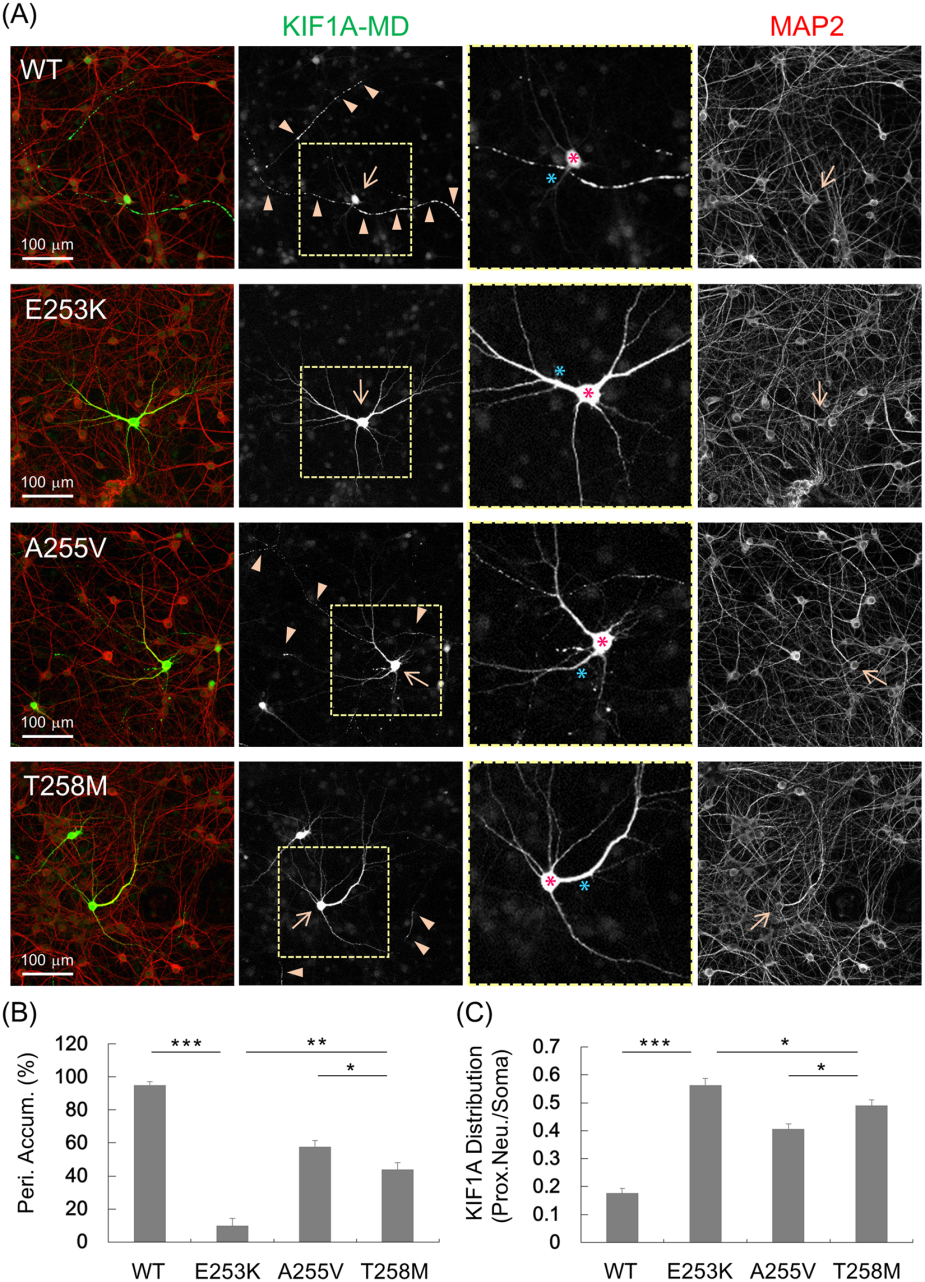
Functional impact of p.T258M mutation on KIF1A motor activity. (**A**) KIF1A-MD-EGFP constructs were expressed in cultured hippocampal neurons and visualized by immunofluorescence staining with anti-EGFP antibody. WT KIF1A-MD accumulated in distal regions of the axons (arrow heads), but KIF1A-MD-T258M showed a considerably reduced peripheral accumulation. The neuronal cell bodies (arrow) and dendrites were visualized by immunofluorescence staining for MAP2. (**B**) Quantification of the peripheral accumulation of KIF1A-MD (mean ± s.e.m.). The distal distribution of KIF1A-MD was analyzed as described in the methods section. Significant decreases are indicated (***p < 0.0001: **p < 0.001: *p < 0.05). (**C**) Quantification of the proximal distribution of KIF1A-MD (mean ± s.e.m.). The proximal distribution of KIF1A-MD was analyzed by measuring the brightness of the proximal neurites (blue asterisk) and the soma (red asterisk) as described in the methods section. Significant decreases are indicated (***p < 0.0001: *p < 0.05).

Using the same strategy, we expressed WT or mutant KIF1A-MD-EGFP (T258M) expression constructs in cultured hippocampal neurons and analyzed their localization in neurites (Fig. 6A). WT KIF1A-MD showed extensive peripheral accumulation (95.2%, 5 trials), but KIF1A-MD-T258M showed considerably reduced distal localization compared with WT (44.0%, 5 trials) (Fig. 6B). Interestingly, the impact of the p.T258M mutation on peripheral accumulation seemed to be intermediate between p.E253K of de novo mutation and p.A255V of autosomal recessive inheritance: KIF1A-MD-E253K (9.7%, 5 trials), KIF1A-MD-A255V (57.7%, 5 trials)^15,20,21^. Transfection of these constructs in HEK293 cells followed by Western blotting indicated that the mutant and WT KIF1A-MD proteins were expressed at similar levels (Supp. Fig. S1).

Previously we have reported that KIF1A-MD proteins with de novo mutations identified from patients with cognitive impairment are abnormally accumulated in the proximal regions of neuronal cell body^15^. Here, the proximal distribution of KIF1A-MD-T258M was increased considerably, as compared with WT proteins: WT KIF1A-MD (0.177, proximal neurite/soma, 6 trials), KIF1A-MD-T258M (0.490, proximal neurite/soma, 7 trials) (Fig. 6C). Similar to the results of the peripheral distribution test, p.T258M mutation had an impact on proximal distribution at levels between those of p.E253K and p.A255V: KIF1A-MD-E253K (0.564, proximal neurite/soma, 6 trials), KIF1A-MD-A255V (0.407, proximal neurite/soma, 6 trials). These results suggest that the p.T258M *KIF1A* mutation identified in the family with autosomal dominant HSP exerts an intermediate impact on *KIF1A* motor activity, at the levels weaker than the p.E253K de novo mutation from cognitive impairment but stronger than p.A255V mutation from autosomal recessive HSP.

### Dominant negative effect of p.Thr258Met mutation on WT KIF1A motor

The p.T258M mutation was identified as a heterozygous mutation, similar to de novo mutations found in patients with developmental delay. A dominant negative effect has been suggested to explain the phenotypes of de novo heterozygous mutations that are more severe than those observed in patients with recessive mutations^15^. Because KIF1A could be converted into a functional dimer, we reasoned that any mutations of motor domain could exert dominant negative effect.

**Figure 7:**
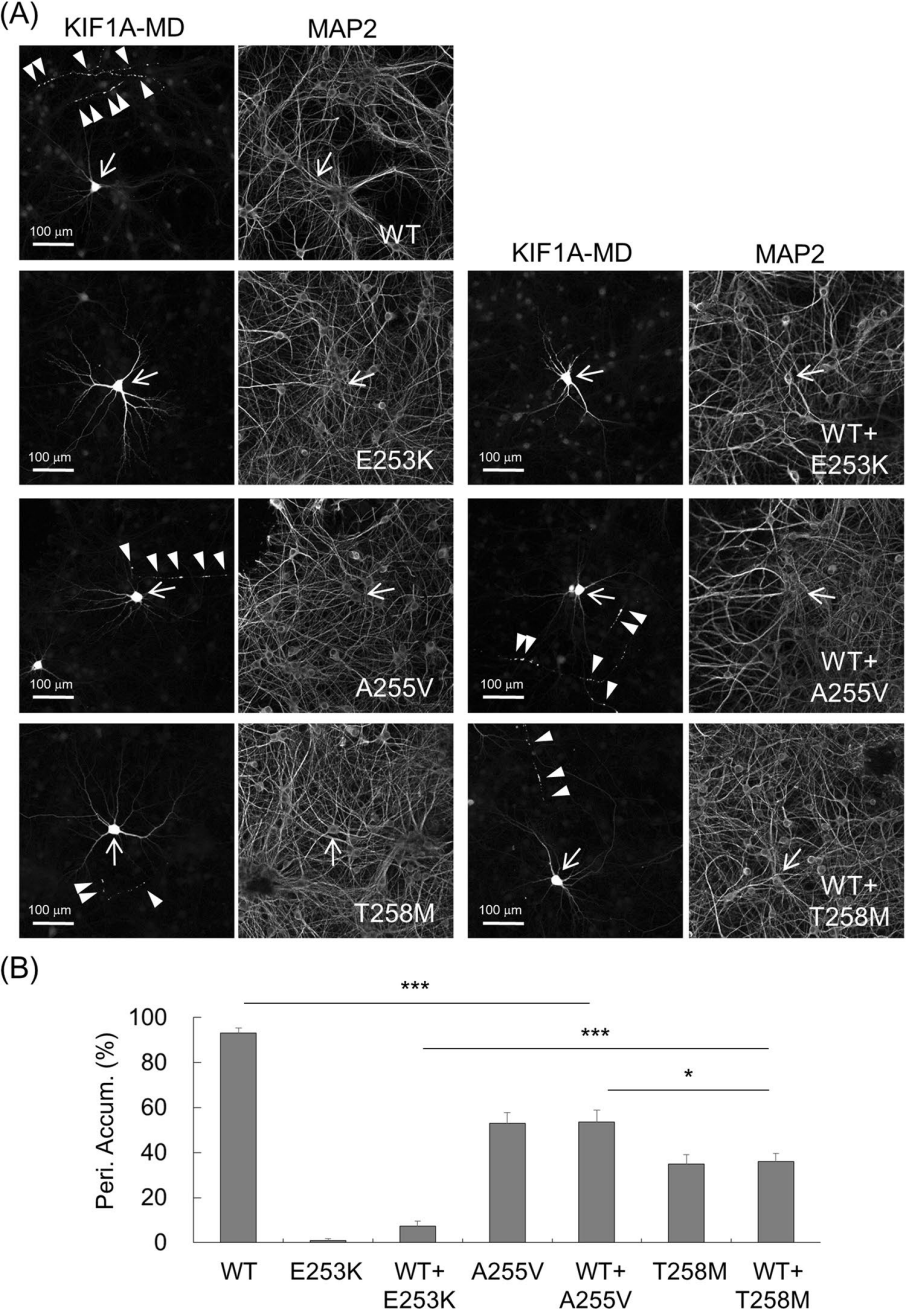
Dominant negative effect of p.T258M mutation on KIF1A motor activity. (**A**) WT KIF1A-MD-EGFP showed considerably reduced peripheral accumulation when co-expressed with KIF1A-MD-T258M (arrow heads). Neuronal cell bodies (arrow) and dendrites were visualized by immunofluorescence staining for MAP2. (**B**) Quantification of the peripheral accumulation of KIF1A-MD proteins (mean ± s.e.m.). The distal distribution of KIF1A-MD was analyzed as described in Methods. Significant decreases are indicated (***p < 0.0001: *p < 0.05).

To this end, the peripheral distribution of WT KIF1A-MD was analyzed in the presence and absence of the co-expression with a mutant KIF1A-MD in hippocampal neurons (Fig. 7A,B). When KIF1A-MD-E253K was co-expressed, the peripheral accumulation of WT KIF1A-MD was dramatically reduced to the level of expression of KIF1A-MD-E253K only: WT KIF1A-MD (93.1%, 8 trials), KIF1A-MD-E253K (0.8%, 8 trials), WT KIF1A-MD with KIF1A-MD-E253K (7.4%, 8 trials). Co-expression of KIF1A-MD-T258M considerably reduced the distal localization of WT KIF1A-MD to levels similar to those of KIF1A-MD-T258M only: KIF1A-MD-T258M (35.1%, 8 trials), WT KIF1A-MD with KIF1A-MD-T258M (35.9%, 8 trials). Interestingly, KIF1A-MD-A255V exerted the dominant negative effect on KIF1A motor activity, but to lesser extent than KIF1A-MD-T258M: KIF1A-MD-A255V (53.24%, 8 trials), WT KIF1A-MD with KIF1A-MD-A255V (53.5%, 8 trials). These results suggest that all the tested *KIF1A* mutations could exert dominant negative effects on WT KIF1A, and the degrees of the dominant negative effects depend on the types of the mutations; strongest in the p.E253K de novo mutation, intermediate in the p.T258M mutation of autosomal dominant inheritance, and weakest in the p.A255V mutation of autosomal recessive inheritance.

## Discussion

In the present study, we identified a novel p.T258M mutation in *KIF1A* affecting mother and three children with spastic paraplegia that is inherited in a type of autosomal dominant manner. Previous studies have identified two cases of a dominantly inherited *KIF1A* variant (p.S69L) from pure HSP^22,23^. However, our patients with the p.T258M mutation in *KIF1A* displayed phenotypes of intellectual disability with language delay, optic nerve hypoplasia, thinning of corpus callosum, periventricular white matter lesion, microcephaly, and epilepsy in addition to spasticity. The inheritance of complicated HSP with intellectual disability has not been reported as far, and our p.T258M *KIF1A* mutation is the first case of autosomal dominant inheritance in familial complicated HSP (Table 2).

**Table 2:** Clinical phenotypes and inheritance patterns of patients with various missense mutations in the motor domain of *KIF1A*.

Mutation	Phenotype	Intellectual Disability	Transmission	Mutation	Phenotype	Intellectual Disability	Transmission
p.S58L	Complicated	Mild	De novo	p.R216C	Complicated	Severe	De novo
p.S69L	Pure spastic	No	Familial AD	p.R216H	Complicated	Moderate	De novo
p.T99M	Complicated	Yes/Severe	De novo	p.R216P	Complicated	Yes	De novo
p.G102D	Complicated	Mild	De novo	p.L249Q	Complicated	Moderate	De novo
p.G102S	Complicated	Mild	Sporadic	p.E253K	Complicated	Severe	De novo
p.V144F	Complicated	Moderate	De novo	p.A255V	Pure spastic**	No	Familial AR
p.R167C	Complicated*	Mild	De novo	p.T258M***	Complicated	Moderate/Mild	Familial AD
p.G199R	Complicated	Severe	De novo	p.R307Q	Complicated	Severe	De novo
p.A202P	Complicated	Yes	De novo	p.R316W	Complicated	Mild	De novo
p.S215R	Complicated	Yes	De novo	p.R350G	Complicated	No	Familial AR

^*^Another case of pure form reported^15,23^.

^**^Another case of complicated form reported^20,21^.

^***^New mutation identified in this study.

Deleterious heterozygous mutations of *KIF1A* in developmental delay have been suggested to act dominant negative effects, and this could explain their phenotype more severe than that those observed in patients with recessive mutations^14,15^. However, the molecular and cell biological evidence for the dominant negative effects could not be obtained because only mutant KIF1A-MD-EGFP proteins have been used to simply monitor protein accumulation at distal and proximal regions of neurites. Here, we attempted new strategy to evaluate dominant negative effects: co-expression of WT KIF1A-MD with mutant KIF1A-MD in cultured hippocampal neurons followed by the evaluation of peripheral distribution. When co-expressed with mutants, WT KIF1A-MD showed dramatically reduced peripheral accumulation (Fig. 7). KIF1A-MD-T258M reduced the peripheral accumulation of WT KIF1A-MD effectively, although to a lesser extent than that exerted by KIF1A-MD-E253K carrying de novo mutation identified in global developmental delay with severe intellectual disability and to a great extent than the KIF1A-MD-A255V with a mutation from autosomal recessive HSP.

It has been suggested that dimerization of KIF1A motors can induce dominant negative effects of heterozygous KIF1A mutations^14,15^. Given that KIF1A can form dimers, autosomal recessive mutations may also exert dominant negative effects although likely more weakly. Our data indeed suggested that all the tested mutations in the motor domain can exert dominant negative effects on KIF1A motor activity: the p.E253K de novo mutation exerted the strongest dominant negative effect, and the p.A255V mutation of autosomal recessive inheritance exerted the weakest dominant negative effect, while the p.T258M mutation of autosomal dominant inheritance exerted an intermediate dominant negative effect.

Each mutation appears to exert various degrees of dominant negative effects according to their locations and charge properties of the mutated amino acids in the motor domain: e.g. acidic to basic one (Glu to Lys) in p.E253K de novo, polar to hydrophobic one (Thr to Met) in p.T258M autosomal dominant, and hydrophobic to hydrophobic one (Ala to Val) in p.A255V autosomal recessive. Notably, the severities of the charge change correlate with the impacts of the mutations on motor activity in the order of p.E253K, p.T258M, and p.A255V mutations.

It is likely that truncating mutations exert more deleterious phenotypes than point mutations. However, nonsense truncating mutations (p.L947Rfs*4 and p.S1758Qfs*7) of *KIF1A* from HSANII with peripheral nerve degeneration resulted autosomal recessive inheritance^18^. Their truncation mutations cause premature stop codons in the middle of the full-length KIF1A or in the C-terminal PH domain through which KIF1A associates with cargoes for synaptic transportation. It has been suggested that cargo-binding to the C-terminal PH domain could be the first step that initiates the dimerization and activation of KIF1A motor^6,7^. Therefore, a *KIF1A* truncation mutant without the PH domain is unlikely to disturb the function of WT KIF1A motor proteins, and exert an enough dominant negative effect to induce an autosomal dominant inheritance.

In conclusion, our data suggest that a novel heterozygous mutation (p.T258M) in *KIF1A* can induce HSP accompanying intellectual disability and this is the first case of familial complicated HSP transmitted in autosomal dominant inheritance.

## Methods

### Subjects and Exome Sequencing Analysis

The study included 4 subjects from a non-consanguineous family from Korea presenting with cognitive impairment, spastic gait, and negative test result for the spastic paraplegia 4 (SPG4) gene. Written informed consent was obtained from the family for the blood samples and the study was approved by the Institutional Review Board (IRB) of the Pusan National University Yangsan Hospital (IRB number: 05–20117–052). All experiments in this study were performed in accordance with the relevance guidelines. Library preparation was performed with a TruSight One Sequencing Panel Kit (Illumina, USA). This panel enriches for approximately 4,800 genes of clinical relevance. Massive parallel sequencing was conducted with a NextSeq. 500 sequencing instrument (Illumina, USA). Local realignment and recalibration were performed using the Genome Analysis Tool Kit (GATK version 3.30). Among the 7,853 called variants, 1,215 remained after filtering for common variants with a minor allele frequency (1%) using multiple population databases (1000 Genomes Project, Exome Variant Server, and Exome Aggregation Consortium). Nucleotide numbering of the mutations herein reflects cDNA numbering with +1 corresponding to the A of the ATG translation initiation codon in the NCBI reference sequence NM_001244008.1, while the amino positions are based on the corresponding NCBI reference sequence NP_001230937.1. Prediction of the effects of the de novo missense *KIF1A* mutations on the protein function were done using PolyPhen-2 (http://genetics.bwh.harvard.edu/pph2/) and SIFT (http://sift.jcvi.org/), and MutationTaster (http://www.mutationtaster.org/), respectively.

### Structural Modeling

Structural modeling of the KIF1A motor with p.Thr258Met mutation was performed using the crystal structure of the KIF1A motor domain in complex with ADP [PDB ID: 1I5S], which has a complete structure of L11, unlike other reported crystal structures of KIF1A with unmodelled L11 structure for the lack of electron density in this region. The p.Thr258Met point mutation was introduced to the KIF1A motor domain using the mutagenesis option in PyMOL, a molecular visualization software^29^. Energy minimization and loop flexible modeling were performed using Modeller software^30^. All structural images were generated using PyMOL^29^.

### Analysis in Cultured Hippocampal Neurons

KIF1A mutation, p.T258M was introduced by site-directed mutagenesis into an expression construct encoding motor domain of mouse KIF1A (aa 1–365) (KIF1A-MD-EGFP)^15^. Primary cultured hippocampal neurons were prepared from rat embryonic brain as described previously^10^. This study was performed in accordance with the regulations outlined by the Korean law. The animal experiment protocols were approved by the Animal Use and Care Committee of Korea Research Institute of Bioscience and Biotechnology (Permit Number: KRIBB-AEC-16138). Animals were sacrificed using CO_2_ gas, and all efforts were made to minimize suffering. Using the calcium phosphate method, cultured hippocampal neurons were transfected with KIF1A-MD-EGFP. Five days after transfection, neurons were fixed and stained with anti-EGFP and anti-MAP2 antibodies followed by fluorescent secondary antibodies. Images were captured by a confocal microscope (LSM800, Zeiss) and analyzed using the MetaMorph software (Universal Imaging), as described previously^15^. Data were acquired from 5~6 trials for each mutant or wild type KIF1A-MD-EGFP and 10~15 images of neurons were collected for each trial. The methods about analyses of the peripheral accumulation and the proximal distribution were described previously^15^. The peripheral accumulation was quantified by calculating the number of neurons that show a distal distribution of the KIF1A-MD-EGFP over the total number of the transfected neurons in each trial. The proximal distribution was analyzed by measuring the brightness of the EGFP signal in the center of the neuronal cell body (soma) and in the segments of neurites (2~3/neuron) extending over 30 μm from the soma as described previously^15^. Then the ratio of the brightness in proximal neurites over the brightness in the soma in 6~8 neurons per trial was calculated. Statistical significance was assessed using the Student’s *t*-test. The dominant negative effects of KIF1A mutants were evaluated by analyzing the peripheral accumulation of wild type KIF1A-MD-EGFP co-expressed with mutant KIF1A-MD-EGFP. For analysis of dominant negative effects, the same amounts of DNA expression constructs were transfected into rat hippocampal neurons, and then neurons were fixed and stained with primary antibodies followed by fluorescent secondary antibodies.

## Electronic supplementary material

Supplementary Information
