# Supplementary material for: Autosomal dominant transmission of complicated hereditary spastic paraplegia due to a dominant negative mutation of KIF1A, SPG30 gene
Source: Sci Rep. 2017 Oct 2;7:12527. doi: 10.1038/s41598-017-12999-9 (PMC5624960; doi:10.1038/s41598-017-12999-9)

Autosomal dominant transmission of complicated hereditary spastic paraplegia due to a dominant negative mutation of *KIF1A*, SPG30 gene

Chong Kun Cheon[1,2*], So-Hee Lim[3], Yoo-Mi Kim[1], Doyoun Kim[4], Na-Yoon Lee[3], Tae-Sung Yoon[3], Nam-Soon Kim[3], Eunjoon Kim[4,5] & Jae-Ran Lee[3*]

[1]Department of Pediatrics, Pusan National University Children's Hospital, Yangsan, Korea. [2]Research Institute for Convergence of Biomedical Science and Technology, Pusan National University Yangsan Hospital, Yangsan, Korea. [3]Rare Disease Research Center, Korea Research Institute of Bioscience and Biotechnology, Daejeon, Korea. [4]Center for Synaptic Brain Dysfunctions, Institute for Basic Science (IBS), Daejeon, Korea. [5]Department of Biological Sciences, Korea Advanced Institute of Science and Technology, Daejeon, Korea.

Figure S1. Expression of KIF1A-MD mutants is comparable to WT KIF1A-MD. (A) WT KIF1A-MD and KIF1A-MD mutants were transfected in HEK293 cells using the calcium phosphate method and the expression of the KIF1A-MD proteins was assessed in the total cell lysates by Western blotting using an anti-EGFP antibody after 2 days of transfection. The levels of expressions of KIF1A-MD mutants were comparable to WT KIF1A-MD. (**B)** The expression of WT KIF1A-MD and KIF1A-MD mutants were confirmed by immunocytochemistry using EGFP antibody in transfected COS1 cells as described in the methods. KIF1A-MD mutants as well as WT KIF1A-MD were evenly distributed throughout the whole cell body.

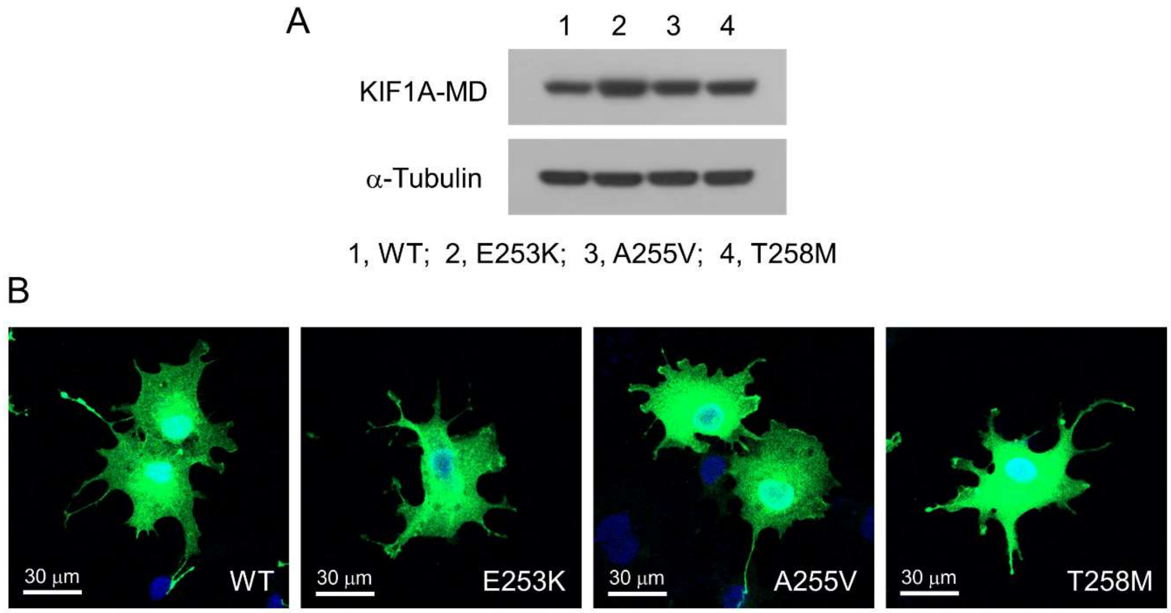

Supplement: Supplementary file 1 — Supplementary Information [file 41598_2017_12999_MOESM1_ESM.pdf]
